# Comprehensive Isomer-Resolved Ganglioside Profiling by HILIC-nanoESI-MS/MS

**DOI:** 10.1021/acs.analchem.6c00380

**Published:** 2026-07-06

**Authors:** Nina Troppmair, Jonas Santol, Mark Baumert, Lukáš Opálka, Alice Assinger, Cristina Coman, Robert Ahrends

**Affiliations:** † Department of Analytical Chemistry, Faculty of Chemistry, University of Vienna, 1090 Vienna, Austria; ‡ Vienna Doctoral School in Chemistry, University of Vienna, 1090 Vienna, Austria; § Department of Vascular Biology and Thrombosis Research, Center of Physiology and Pharmacology, 550100 Medical University of Vienna, 1090 Vienna, Austria; ∥ Department of Surgery, HPB Center, Vienna Health Network, Clinic Favoriten and Sigmund Freud Private University, 1100 Vienna, Austria; ⊥ Division of Hepatobiliary and Pancreas Surgery, Department of Surgery, Mayo Clinic, Rochester, Minnesota 55905, United States; # 321009 Advion Ltd, Harlow Enterprise Hub, Harlow CM20 2NQ, United Kingdom; ∇ Department of Organic and Bioorganic Chemistry, Faculty of Pharmacy, Charles University, 500 03 Hradec Králové, Czech Republic; ○ Ludwig Boltzmann Institute of Cardiovascular Research, 1090 Vienna, Austria

## Abstract

Gangliosides are structurally complex glycosphingolipids that regulate cellular signaling via interactions with extracellular binding partners and by influencing protein function within the membrane. Their comprehensive analysis remains analytically challenging, in part due to low endogenous abundance, extensive structural diversity, and the frequent occurrence of isomeric species arising from both glycan and ceramide moieties. Here, we present a hydrophilic interaction liquid chromatography (HILIC) nanoelectrospray ionization tandem mass spectrometry (nanoESI-MS/MS) method specifically designed to achieve isomer-resolved analysis and molecular species-level quantification of gangliosides. To improve characterization of low-abundance fatty acyl fragments, the workflow was extended by combining nanoESI with online fraction collection and subsequent direct infusion. The method demonstrated stable chromatographic performance, high sensitivity, and reproducible quantitative results across ten mouse tissues. Application of the workflow enabled quantification of 80 ganglioside molecular species belonging to 18 subclasses and revealed pronounced tissue-specific differences in subclass distribution and ceramide composition. Overall, this study establishes a robust and sensitive platform for comprehensive ganglioside analysis, providing new opportunities to investigate ganglioside diversity, regulation, and function in complex biological systems.

## Graphical abstract

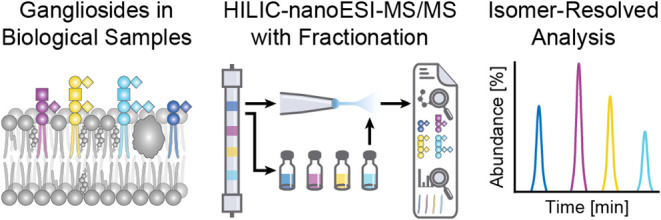

## Introduction

Gangliosides are lipids belonging to the acidic glycosphingolipids, a subclass of sphingolipids, which are one of the eight lipid categories defined by the LIPID MAPS classification.^1^ In general, sphingolipids consist of a sphingoid base (also known as long-chain base; LCB) which is linked via an amide bond to an alkyl chain (fatty acid; FA). The subclass of gangliosides is characterized by the presence of a headgroup (HG) built by mono- or polysialylated oligosaccharides.^2–6^ The structural diversity of ganglioside species arises from variations in their sphingoid base, N-linked fatty acyl chains, and glycan moieties. Specifically, like for other sphingolipids the heterogeneity of the ceramide backbone stems from differences in chain length, saturation and hydroxylation. This complexity is further increased by the composition of oligosaccharide HG, which may contain various sugars, including neutral monosaccharides such as glucose (Glc) and galactose (Gal), with the potential for further modification by additional neutral sugars like N-acetylgalactosamine (GalNAc), N-acetylglucosamine (GlcNAc), and fucose (Fuc). Furthermore, gangliosides feature ionizable functional groups such as sialic acid residues, with variations in the number and position of sialic acids contributing significantly to the great diversity of the ganglioside lipidome.^2–5^ The trivial name sialic acid is generally used for all derivatives of neuraminic acid. Among these, N-acetylneuraminic acid (Neu5Ac) is the most commonly found sialic acid in humans, while N-glycolylneuraminic acid (Neu5Gc) is abundant in many nonhuman species, such as mice.^2,4,5^

Capturing this molecular diversity in complex biological samples therefore requires sophisticated analytical methods. Traditional analytical methods, such as thin-layer chromatography (TLC), necessitate purification and preconcentration steps, making them time-consuming and only suitable for analyzing a limited number of samples with large sample volumes. Additionally, these methods lack the specificity required to monitor the structural diversity of gangliosides resulting from the lipophilic ceramide and a hydrophilic oligosaccharide unit.^3,7^ Most commonly biphasic extraction systems based on chloroform, methanol, and water, such as the Svennerholm,^8^ Folch^9^ and Bligh–Dyer^10^ methods, have been employed in ganglioside analyses.^2,3,11,12^ Further, monophasic extraction strategies have also been applied.^13,14^ In addition, solid-phase extraction (SPE) techniques have been used for the enrichment and purification of gangliosides prior to analysis.^12,15^ Bulk ganglioside content has been assessed using immunochemical and immunohistochemical methods as well as TLC.^16,17^ More recently, reversed-phase liquid chromatography (RPLC), and hydrophilic interaction liquid chromatography (HILIC) coupled with tandem mass spectrometry (MS/MS) has emerged as a powerful tool for the characterization and quantification of gangliosides in biological samples.^2,3,7,18^ Alternatively, direct infusion has proven suitable for analyzing gangliosides in limited sample amounts.^11^ However, these methods remain partly insufficient to fully resolve the ganglioside lipidome due to limitations in their separation capabilities and the inability to create diagnostic fragment ions, which would allow to discriminate isomeric species.^3^ Advanced techniques such as ultraviolet photodissociation mass spectrometry^19^ and ozonolysis^20^ gained importance due to the potential to provide deeper structural information. These approaches are, however, limited by the availability of such instrumentation.

In this study, we present a method using HILIC with nanoelectrospray ionization (nanoESI) MS/MS, optimized to separate HG isomers and obtain structural information on species-specific LCB and FA moieties. To further enhance structural characterization, this approach is complemented by an online fractionation strategy, enabling the collection of fractions for subsequent infusion-based MS/MS analysis. This combined workflow allows detailed elucidation of ceramide compositions while maintaining headgroup resolution, which is essential given that ganglioside function is determined by the precise structure of both the glycan headgroup and the ceramide moiety.^4,6^ The glycan composition and linkage pattern define specific interactions with lectins, antibodies, and other carbohydrate-binding proteins, as well as carbohydrate-carbohydrate interactions at the cell surface.^21–23^ In contrast, variations in lipid acyl chains influence fundamental biophysical properties of membranes, such as bilayer thickness, bending rigidity, lateral packing, and spontaneous curvature, thereby modulating interactions with neighboring lipids and membrane proteins.^24–26^ Isomer-resolved, molecular species-level analysis is therefore essential to understand how structurally distinct gangliosides exert specific biological functions in complex membranes.

## Experimental Section

### Materials

All chemicals used for solvent preparation were purchased from Sigma-Aldrich (Steinheim, Germany), Biosolve (Valkenswaard, The Netherlands), Honeywell (Seelze, Germany), or Merck (Darmstadt, Germany). Lipid standards used in this study were obtained from Avanti Polar Lipids (Alabaster, AL, USA) and Cayman Chemical (Ann Arbor, MI, USA). Additional experimental details are provided in the Supporting Information.

### Sample Preparation

Mouse tissues were homogenized by bead-based disruption in MeOH/H_2_O (1:1, v/v) using a Precellys Evo homogenizer (Bertin Technologies, Montigny-le-Bretonneux, France). For each sample, an aliquot corresponding to 5 mg wet tissue was subjected to lipid extraction following a slightly modified protocol based on Coman et al.^27^ (see Supporting Information for details). This procedure enabled the recovery of gangliosides from the aqueous phase, while less polar sphingolipids were obtained from the organic phase. Prior to extraction, each sample was spiked with seven ganglioside internal standards, as well as class-specific internal standards for all other analyzed sphingolipid classes.

### LC-nanoESI-MS/MS

Ganglioside analysis was conducted using a Vanquish Horizon UHPLC system (Thermo Fisher Scientific, Germering, Germany) connected to a TriVersa NanoMate ion source (Advion BioSciences, Ithaca, NY, USA) coupled to an Exploris 240 mass spectrometer (Thermo Fisher Scientific, Bremen, Germany). Samples were injected at a volume of 5 μL onto an ACQUITY Premier Glycan BEH Amide column (150 mm × 2.1 mm, 1.7 μm; Waters, Wexford, Ireland) equipped with an integrated VanGuard FIT guard column. The autosampler was maintained at 10 °C, and the column oven was set to 40 °C. Mobile phase A consisted of 95% 15 mM ammonium acetate at pH 6.1 and 5% IPA, while mobile phase B was 100% ACN. Chromatographic separation was achieved using a 22 min gradient at a constant flow rate of 0.2 mL/min (see Supporting Information for details). The injection needle was automatically washed with a mixture of ACN/MeOH/H_2_O (1/1/1, v/v/v) prior to each injection. The UHPLC flow was split after the column such that 500 nL/min were directed to the mass spectrometer, while the remaining flow (199.5 μL/min) was either collected online into fractions or diverted to waste during wash steps. The ionization voltage was set to −1.7 kV and data were acquired in negative ion mode. The acquisition method was divided into two sequential segments with different full-scan *m*/*z* ranges and isolation windows, tailored to the ganglioside subclasses eluting within each time window. In both segments, full-scan acquisition was followed by data-dependent MS/MS of the 20 most intense precursor ions using a normalized collision energy (nCE) of 30%. Additional instrument parameters, as well as the methods used for analysis of other sphingolipid classes in murine tissues, are described in the Supporting Information.

### Direct Infusion MS and MS/MS of Fractions

For direct infusion experiments the collected fractions were infused directly from 96-well plates using a TriVersa NanoMate ion source (Advion BioSciences, Ithaca, NY, USA) coupled to an Exploris 240 mass spectrometer (Thermo Fisher Scientific, Bremen, Germany). To increase the number of gangliosides identified at the molecular species level, the nCE was set to 53%, promoting fragmentation of the ceramide moiety. Detailed instrument settings are provided in the Supporting Information.

### Data Processing

Data acquisition was performed using Thermo Scientific Xcalibur (version 4.7.102.25) and the Orbitrap Exploris 240 Tune Application (version 4.4.550.18; Thermo Fisher Scientific) for LC-nanoESI-MS/MS and shotgun lipidomics experiments, respectively. Skyline (version 25.1.0.142)^28^ was used for visualization and manual integration of LC-MS data. Isotopic correction was applied using a custom script written in R (RStudio version 2024.12.0 + 467; RStudio Team). Raw data from shotgun lipidomics experiments were processed using LipidXplorer (version 1.2.9)^29,30^ with self-written molecular fragmentation query language (MFQL) query files. Postacquisition data processing was automated using the software tool Konstanz Information Miner (KNIME; version 5.2.5).^31^ Figures were prepared using BioRender.com, OriginPro (Version 9.8.0.200; OriginLab Corporation), and ChemDraw (Version 20.0.0.41; PerkinElmer Informatics).

## Results and Discussion

### Method Development and Optimization

Quantifying gangliosides in complex biological matrices is challenged by the coexistence of many structurally related species that differ in glycan and ceramide composition and span a wide dynamic range across tissues. To address this, we developed a HILIC-nanoESI-MS/MS method specifically optimized for the separation of ganglioside isomers originating from both the carbohydrate and lipid moieties.

Out of three tested HILIC columns, an amide-bonded stationary phase was selected due to its strong retention of highly polar analytes and its suitability for resolving structurally similar glycosphingolipids. Method performance was evaluated using a total ganglioside extract from porcine brain. Using an optimized LC method with a total gradient time of 22 min, gangliosides with 32 distinct glycan structures were detected (Figure 1A). These species differ in sialylation state and include additional modifications such as acetylation and fucosylation, while sharing the ceramide composition 18:1;2/18:0;0. Importantly, the optimized HILIC conditions enabled baseline separation of glycan-derived isomers, exemplified by the resolution of GD1a, GD1b, and GD1c. However, for more complex gangliosides and species with additional modifications such as acetylation, the structures shown may represent only one of several possible isomeric forms.

**Figure 1:**
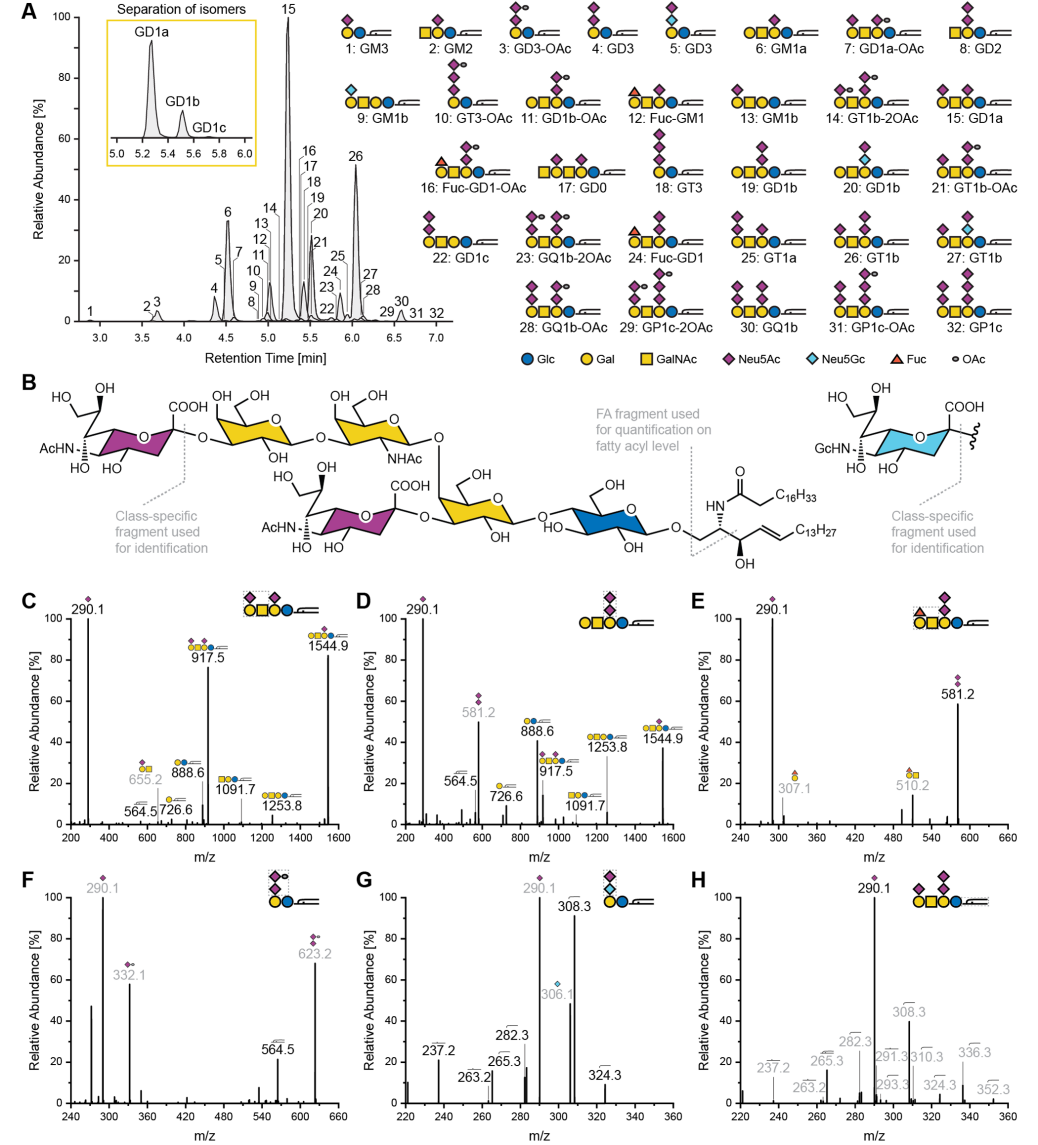
LC-MS/MS method for the analysis of gangliosides at molecular species level. (A) HILIC separation of 32 ganglioside subclasses detected in porcine brain extract. The method enables separation of glycan-based isomers, such as GD1a, GD1b, and GD1c (fucosylated and acetylated structures are examples and not unique isomers). (B) MS/MS fragmentation provides complementary structural information. Glycan-derived fragments, including characteristic sialic acid ions, confirm ganglioside identity, while fatty acyl (FA)-derived fragments give information on the ceramide moiety. (C–G) Additional glycan fragments allow further annotation of isomeric headgroups. (H) FA- and long-chain base (LCB)-derived fragments enable quantification at the molecular species level.

Quantification was performed at the MS1 level using class-specific internal standards when available, or structurally similar analogues otherwise. Annotation of lipids as gangliosides required the presence of diagnostic sialic acid fragments at *m*/*z* 290.1 (Neu5Ac) or *m*/*z* 306.1 (Neu5Gc) in the corresponding MS/MS spectra. MS/MS data were subsequently used to estimate the relative abundance of individual isomers differing in their ceramide composition, and these ratios were applied to the total MS1-derived signal to obtain species-specific concentrations (Figure 1B).

As GD1a and GD1b are among the most abundant gangliosides in porcine brain, they were used as benchmark species to demonstrate the successful chromatographic separation of structural isomers. While GD1a 36:1;2 (*m*/*z* 917.5) yields the characteristic fragment ion at *m*/*z* 655.2, only GD1b 36:1;2 (*m*/*z* 917.5) exhibits the diagnostic NeuAc–NeuAc fragment at *m*/*z* 581.2 (Figure 1C,D). Additional fucosylation of this species, giving Fuc-GD1 36:1;2 (*m*/*z* 990.5) was associated with characteristic fragment ions at *m*/*z* 510.2 and *m*/*z* 307.1 (Figure 1E). In contrast, acetylation of one of the two sialic acids, like in GD3-OAc 36:1;2 (*m*/*z* 755.9) produced a fragment at *m*/*z* 332.1. Under these conditions, the previously observed NeuAc–NeuAc fragment at *m*/*z* 581.2 was no longer detected. Instead, a fragment at *m*/*z* 623.2 was observed, consistent with the mass shift introduced by acetylation (Figure 1F). The fragmentation behavior of a ganglioside detected at *m*/*z* 742.9 is shown in Figure 1G. Although this species was initially annotated as GD3 36:1;3, the expected FA-derived fragment was not observed. Instead, fragments corresponding to Neu5Gc (*m*/*z* 306.1) and Neu5Ac (*m*/*z* 290.1) were detected, indicating that the species was more likely GD3 18:1;2/18:0;0 (*m*/*z* 742.9) containing both sialic acid variants. As isomers arising from the ceramide moiety of gangliosides are not chromatographically resolved under HILIC conditions, MS/MS-derived fragment ions are essential for their differentiation. Figure 1H illustrates this for GT1b 38:1;2 (*m*/*z* 1077.0), for which the MS/MS spectrum revealed two LCB- and four FA-derived fragment ions. These fragments correspond to the coeluting isomeric species GT1b 18:1;2/20:0;0 and GT1b 20:1;2/18:0;0. In negative ion mode, the most abundant FA-related fragment was observed as FA + C_2_H_3_N.

Collision energy (CE) was systematically optimized for ganglioside standards representing eight distinct ganglioside classes to enable detection of both class-specific glycan fragments and ceramide-derived ions required for molecular species annotation. Spectra shown in Figure 1C–F were acquired at low CE, where fragmentation was dominated by sequential loss of glycan residues, yielding diagnostic sialic acid fragments used to confirm ganglioside identity. In contrast, higher CE settings were applied for Figure 1G–H, promoting fragmentation of the ceramide backbone and the formation of FA-derived ions. The underlying CE-dependent fragmentation behavior is examined in more detail in Figure 2A, which indicates that optimal fragmentation of the glycan-derived fragment at *m*/*z* 290.1 occurs at a nCE of approximately 30%, whereas maximal intensity of the most abundant FA-derived fragment is achieved at nCEs around 50%. This complementary fragmentation behavior formed the basis for ganglioside identification and quantification throughout the study.

**Figure 2:**
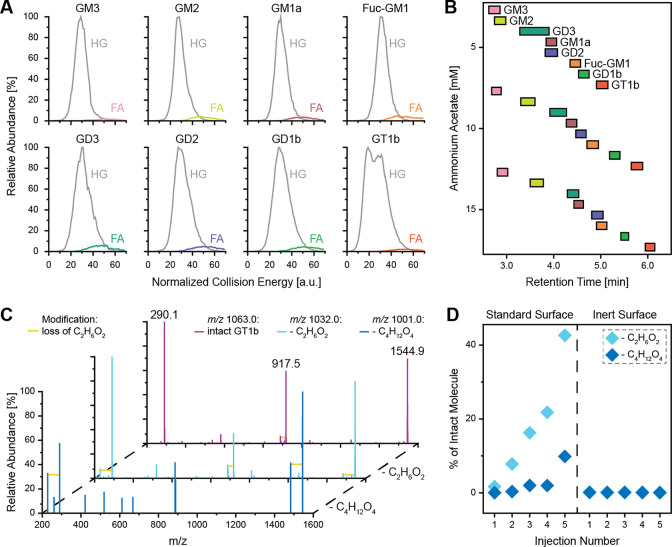
Method optimization for ganglioside analysis. (A) Normalized breakdown curves of eight ganglioside standards illustrating collision energy (CE)-dependent fragmentation. Low CE favors formation of diagnostic sialic acid fragments (*m*/*z* 290.1 for Neu5Ac and *m*/*z* 306.1 for Neu5Gc), whereas higher CE promotes generation of fatty acyl (FA)-derived fragments required for molecular species-level annotation. (B) Effect of ammonium acetate concentration on chromatographic separation. (C) MS/MS spectra highlighting sialic acid modifications manifested as sequential losses of C_2_H_6_O_2_ observed during method optimization. (D) Replacement of a conventional column with a stationary phase featuring an inert surface eliminates modification artifacts and restores signal stability.

We next optimized the mobile phase composition. Previous studies have highlighted the importance of volatile organic salts for HILIC-based glycosphingolipid separations, with ammonium acetate at approximately pH 6.1 reported as optimal.^2,3^ Consistent with these findings, increasing ammonium acetate concentrations improved peak shape but resulted in reduced signal intensity. To identify suitable buffer conditions for the chromatographic setup used here, eight ganglioside standards were analyzed across a range of ammonium acetate concentrations. As shown in Figure 2B, higher salt concentrations led to narrower peaks and improved chromatographic resolution, reflected by increased retention and enhanced separation of the tested gangliosides. Specifically, the retention time difference between the earliest- and latest-eluting standards, GM3 and GT1, increased from 2.3 min at 5 mM to 3.1 min at 15 mM ammonium acetate. The increased overlap observed between the subclasses GD3 and GM1, as well as between GD2 and Fuc-GM1, was not considered detrimental, as it concurrently reduced coelution with other ganglioside subclasses among the approximately 30 commonly detected species for which no internal standards are currently available.

During method optimization, reproducibility issues were observed following column equilibration at the start of new sample sequences. Signal intensities, particularly for more complex gangliosides containing multiple sialic acid residues, progressively decreased, while additional peaks corresponding to mass losses of C_2_H_6_O_2_ from the intact precursor emerged. MS/MS analysis indicated that these mass shifts affected both the diagnostic sialic acid fragment at *m*/*z* 290.1 and higher-mass fragments corresponding to the loss of one (*m*/*z* 917.5, doubly charged) or two (*m*/*z* 1544.9) sialic acid residues from the precursor (Figure 2C), suggesting modification of individual sialic acid moieties. To mitigate these effects, which were presumed to arise from interactions between the analytes and active surfaces within the chromatographic system, the column was replaced with an otherwise identical stationary phase featuring an inert surface. This modification fully restored signal stability and repeatability (Figure 2D), underscoring the importance of minimizing metal surface interactions for reliable ganglioside analysis.

### Establishment of Final Setup

As the primary aim of this study was to obtain isomer-resolved information for both the glycan and ceramide moieties of gangliosides, sufficient intensity of FA-derived fragment ions was required for confident molecular species-level annotation and quantification. While the optimized HILIC separation provided excellent isomer resolution, the resulting narrow chromatographic peaks and nearly simultaneous elution of entire subclasses limited the number of MS/MS spectra that could be acquired per analyte in data-dependent acquisition (DDA) mode. Data-independent acquisition (DIA) was not a viable alternative in this context due to the extensive coelution of structurally related isomers as well as isotopes, causing extensive spectral complexity. To address this limitation, we evaluated an alternative analytical configuration combining a chip-based nanoESI ion source with online fraction collection.

As illustrated, samples obtained from the aqueous phase of a biphasic extraction (Figure 3A) were injected onto the previously mentioned HILIC column using conventional HPLC at a flow rate of 200 μL/min (Figure 3B). Downstream of the column, the eluent was split using an automated ion source platform (Figure 3C). This configuration directed 500 nL/min to the mass spectrometer, while the remaining 199.5 μL/min were collected online into fractions. Fractions of interest were identified during the online LC-MS/MS analysis and were subsequently reanalyzed by direct infusion from the collection plate (Figure 3D). During the LC-MS/MS run, a low nCE of 30% was applied to confirm subclass-specific glycan structures within each fraction. Subsequent direct infusion was performed at a higher nCE of 53% to generate FA-derived fragment ions required for molecular species-level annotation. The additional MS/MS information obtained from direct infusion was integrated with the chromatographic data to generate the final data set (Figure 3E). Prior to normalizing the lipid species to their internal standard, isotopic correction was applied (Figure 3F). Quantification was based on MS1 signal intensities, and relative contributions of individual species were derived from MS2 intensity ratios. Importantly, since all lipids of one subclass are contained in the same fraction, subclass assignment is already defined during the online LC-MS/MS analysis based on retention time and headgroup fragmentation. This enables the use of higher nCE required for FA fragmentation, while only diagnostic sialic acid fragments are needed for confirmation of ganglioside identity.

**Figure 3:**
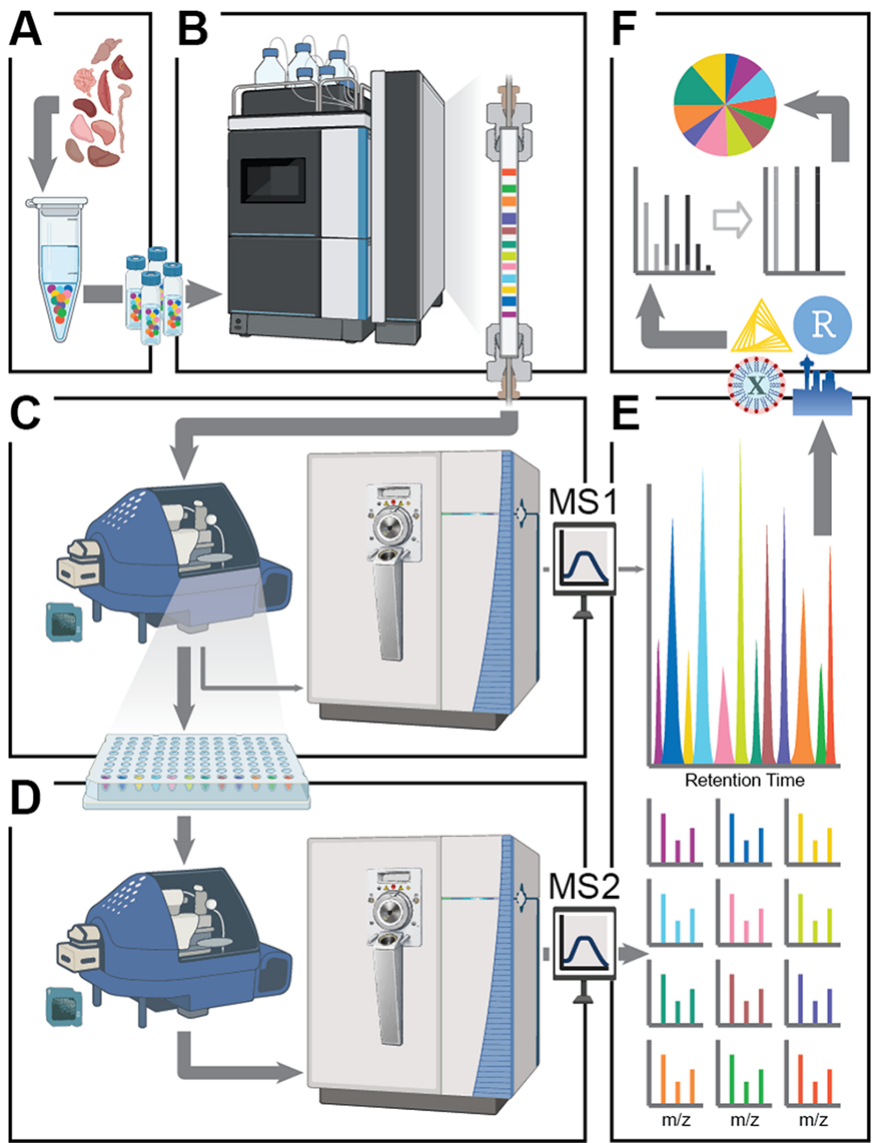
Schematic of the final HILIC-nanoESI-MS/MS setup with online fraction collection. (A) Samples from the aqueous phase of a biphasic extraction (B) are injected onto a HILIC column at 200 μL/min. (C) The eluent is split using an automated ion source platform, delivering 500 nL/min to the MS while the remainder is collected in a 96-well plate. (D) Fractions are subsequently reanalyzed by direct infusion to obtain fatty acyl (FA) fragments, (E) which are combined with the LC-MS/MS data to enable molecular species-level annotation. (F) Data analysis involves peak integration, isotopic correction and normalization to the internal standard.

Overall comparable performance between conventional ESI and nanoESI setups was observed based on signal intensities of ganglioside species with a ceramide composition of 36:1;2 across the 32 subclasses detected in porcine brain extract (Figure 4A). While certain subclasses exhibited higher intensities with one or the other configuration, the strong linear correlation between both data sets (*R* ^2^ = 0.978) indicates highly consistent responses. The close agreement between the linear fit and the identity line further demonstrates the overall comparability of both approaches, while revealing a slight bias toward higher signal intensities for nanoESI, particularly for lower abundant subclasses. Considering the pronounced difference in flow rates (200 μL/min versus 500 nL/min), these results underscore the substantially increased ionization efficiency of the nanoESI setup.

**Figure 4:**
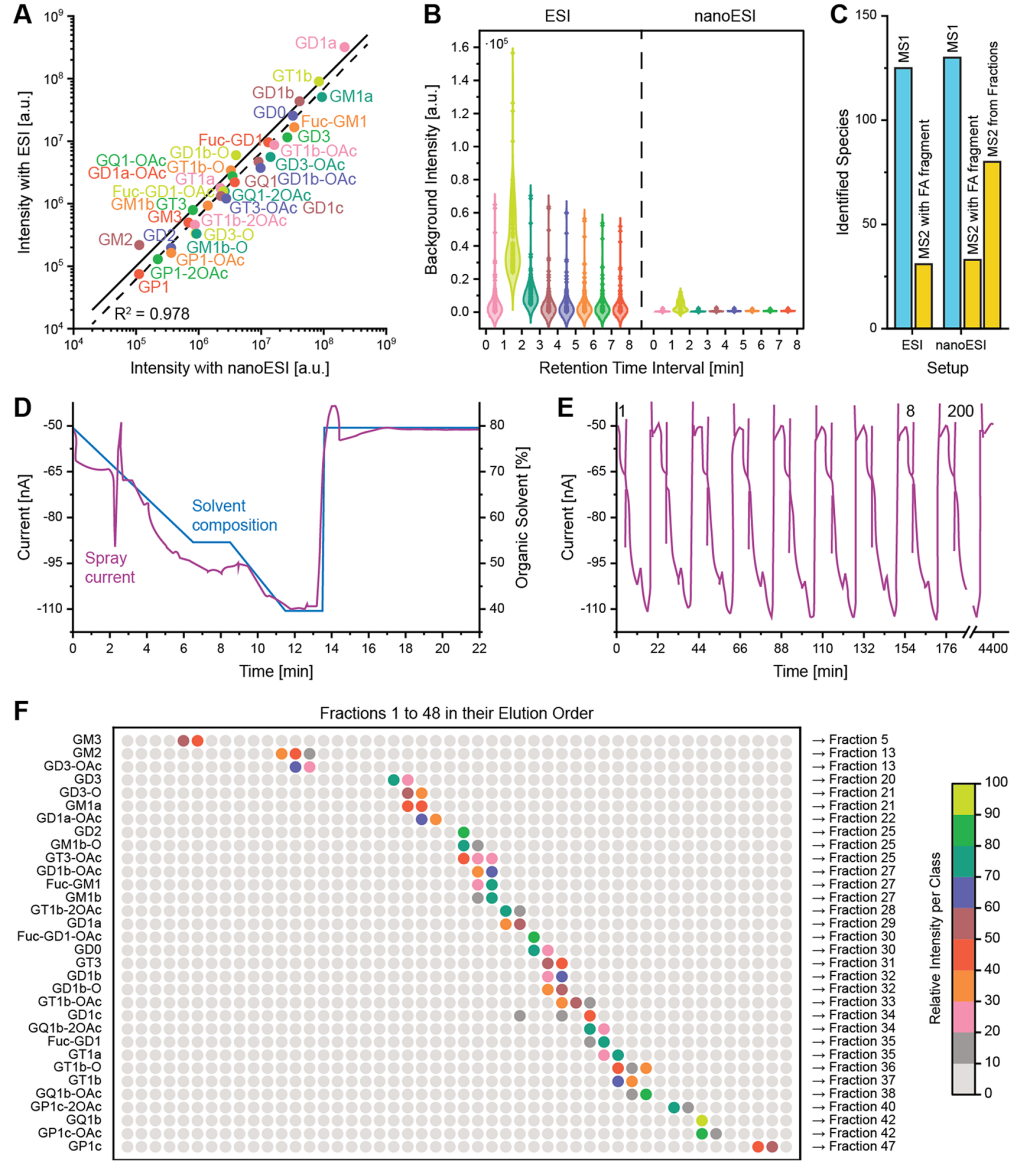
Comparison of conventional ESI and nanoESI setups and evaluation of final method. (A) Signal intensities of ganglioside species with ceramide composition 36:1;2 across 32 subclasses detected in porcine brain extract for conventional ESI and nanoESI setups. The dashed line represents the linear regression, while the solid line indicates equal signal response between both ionization methods. (B) Comparison of chemical background in blank injections for both setups, demonstrating lower background with nanoESI. (C) Number of quantified precursors and species with FA information for both setups. Direct infusion of collected fractions significantly increases the number of gangliosides quantified at the molecular species level. (D) Influence of solvent composition on spray current. (E) Stability of the spray current across 200 consecutive injections using optimized conditions. (F) Example of fraction collection across the chromatographic run (48 fractions), preserving separation of glycan isomers for subsequent FA analysis.

Moreover, analysis of blank injections demonstrated a markedly reduced chemical background with the final configuration (Figure 4B). Using identical solvents, MS1 signal intensities across the entire mass range were more than 1 order of magnitude lower for the nanoESI setup. This substantially reduced background was likewise observed in biological sample injections, resulting in improved signal-to-background ratios. The elevated background observed between minutes 1 and 2 of the gradient corresponds to the injection peak eluting within this interval.

In addition to reduced chemical noise, the nanoESI setup led to reduced adduct heterogeneity, with a single predominant adduct species accounting for more than 90% of the normalized peak area across all eight ganglioside standards (Figure S1).

As shown in Figure 4C, the number of quantified precursors and ganglioside species for which FA information could be obtained was slightly higher using the nanoESI setup compared to conventional ESI. Importantly, reanalysis of collected fractions by direct infusion resulted in a substantial increase in the number of gangliosides quantified at the molecular species level. In total, the final method enabled identification of 80 gangliosides belonging to 27 subclasses at the molecular species level in porcine brain extract, which is commonly used as a reference matrix in ganglioside lipidomics. An additional 50 species could be annotated based solely on exact mass and retention time. To the best of our knowledge, this represents the highest number of ganglioside species reported to date in this reference mixture.^3,19,32^

When employing the automated nanoESI ion source platform, spray stability becomes a critical parameter. As shown in Figure 4D, the spray current is strongly influenced by the mobile phase composition. In line with the preceding optimization experiments shown in Figure 2B, three buffer concentrations were evaluated, namely 5, 10, and 15 mM ammonium acetate. Spray stability was markedly improved at 15 mM ammonium acetate. At lower salt concentrations, particularly 5 mM ammonium acetate, spray stability was insufficient and spray loss occurred after around four injections. To further enhance spray robustness, additional parameters including the solvent composition of the mobile phase, the split ratio and ion transfer tube temperature were optimized. Collectively, these adjustments resulted in a highly stable spray current across numerous injections, as exemplified by eight consecutive runs shown in Figure 4E. In practice, the optimized setup enabled analysis of more than 200 biological samples using a single nanoESI nozzle. Consistent spray performance across different chip batches was further confirmed by comparable spray currents and chromatographic profiles from replicate injections of a porcine brain extract (Figure S2).

Finally, the fraction collection method was optimized to preserve chromatographic separation of glycan isomers, so that species differing only in glycan structure were collected in separate fractions. This allowed FA-derived information to be unambiguously assigned to the corresponding glycan isomer. In the example shown in Figure 4F, 48 fractions were collected across the chromatographic run. The method can be readily adapted to collect only fractions containing eluting analytes, thereby reducing the number of filled wells and sample handling.

### Method Validation

To assess the suitability of the developed HILIC-nanoESI-MS/MS method for the analysis of gangliosides in complex biological matrices, key method characteristics were evaluated for seven ganglioside standards across ten different mouse tissues (brain, heart, kidney, liver, large intestine, lung, muscle, small intestine, spleen, and stomach). Given the extensive structural diversity of gangliosides and their tissue-dependent abundance, robust chromatographic performance and reproducible MS response are essential.

Retention time stability was first assessed, as consistent chromatographic behavior is critical for reproducible fraction collection and subsequent MS-based analysis. Across all ganglioside standards and tissues, retention time variability was remarkably low, with relative standard deviations (RSD) remaining below 0.4% or 0.01 s across six samples measured on different days (Figure 5A). In average, the smallest retention time shift across all tissues was observed for GD1 (0.1%), whereas the largest deviation was detected for GM3 (0.2%), demonstrating that the shifts were slightly higher in the beginning compared to the end of the gradient.

**Figure 5:**
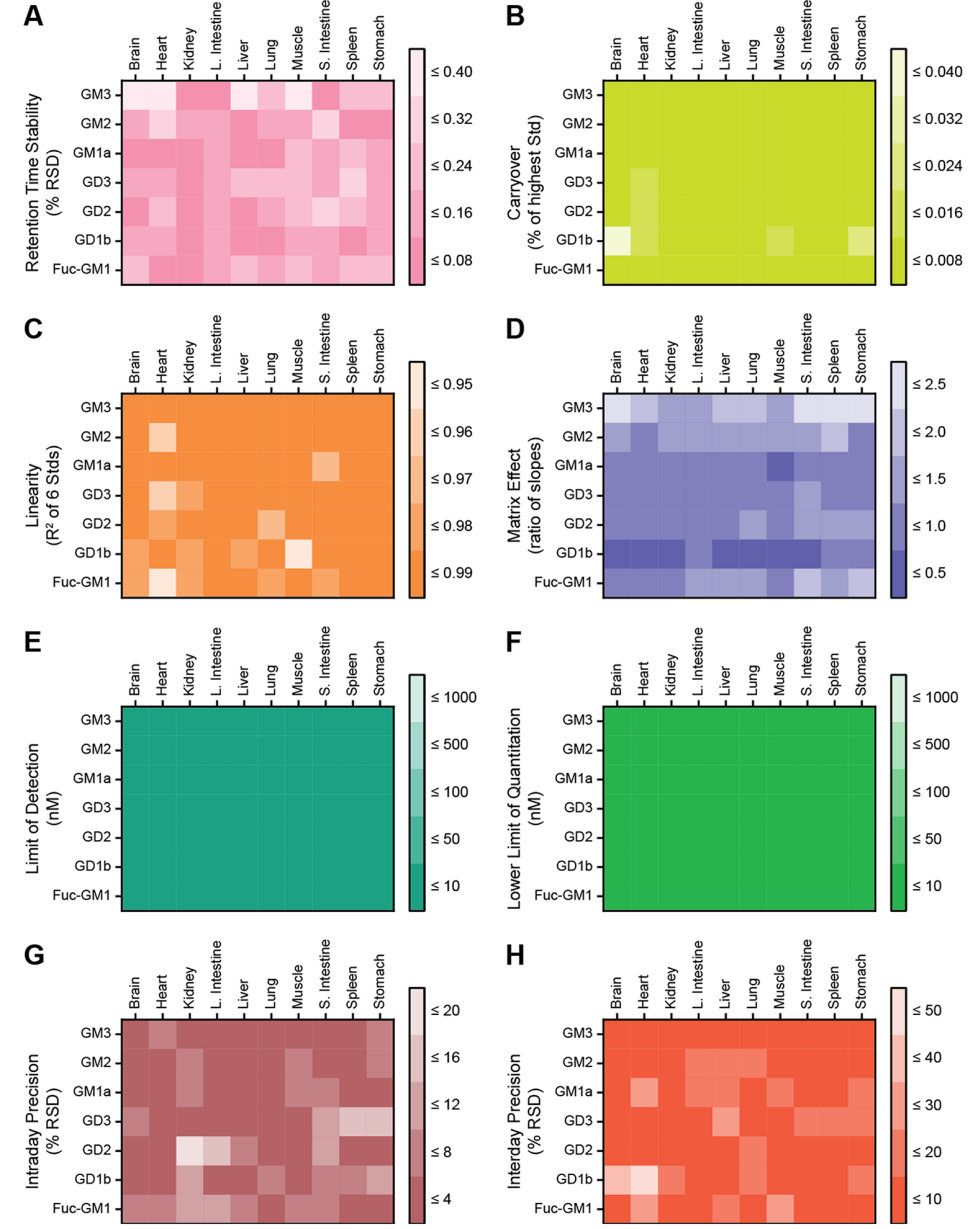
Method characterization across ten mouse tissues. (A) Retention time stability, expressed as relative standard deviation (RSD) across six injections. (B) Carryover assessed by blank injections following the highest calibrator. (C) Linearity of calibration curves based on six concentration levels spanning 3 orders of magnitude. (D) Matrix effects expressed as the ratio of calibration curve slopes obtained in matrix and solvent. (E, F) Limits of detection (LOD) and lower limits of quantification (LLOQ). (G, H) Intraday and interday precision, expressed as RSD.

Carryover was evaluated by injecting blank samples following the highest calibrator for each ganglioside standard and mouse tissue. For all analytes, carryover remained low and did not exceed 0.04% of the preceding signal (Figure 5B), indicating that the method is suitable for sequential analysis of samples spanning a wide range of ganglioside concentrations.

Linearity was assessed across the investigated concentration ranges for all seven ganglioside standards in each tissue. Calibration curves covered each standard spiked at six different concentrations, spanning 3 orders of magnitude, with in average coefficients of determination (*R* ^2^) ≥ 0.99 (Figure 5C). Minor deviations from ideal linearity were observed especially for the later eluting subclasses Fuc-GM1 and GD1, but overall linear performance was maintained across the diverse biological matrices investigated.

Matrix effects were evaluated for each tissue by comparing analyte responses of each standard in matrix to those obtained in neat solvent. For most tested ganglioside standards, matrix effects were moderate, however, a stronger signal enhancement was observed at the beginning of the gradient for GM3, while suppression was most significant for the latest eluting standard GD1 (Figure 5D). The most pronounced matrix effect was observed for GD1 in heart tissue, which is likely attributable to coeluting endogenous components. Importantly, the use of class-specific or structurally similar internal standards effectively compensated for matrix-dependent signal variations, enabling robust quantification across tissues.^33^ With only seven internal standards being available during this study, the more than 30 ganglioside subclasses are by far not covered, however, the standards span nicely over the entire retention time range and enable correcting for matrix effects, which are mostly dependent on the elution time, as observed, even if it is not a class-matched internal standard.

Method sensitivity was characterized by determining limits of detection (LOD) and lower limits of quantification (LLOQ) for all ganglioside standards in each matrix. At the lowest point of the calibration curve, which was 10 nM (corresponding to 0.05 pmol on the column) all standards in all tested tissues were still quantifiable. Thus, the LOD of every standard is below 10 nM, making the method suitable for the analysis of gangliosides with low endogenous abundance (Figure 5E,F). It should be noted that the LOD and LLOQ here are not only referring to the quantification at MS1 level, as performed for this study, but also consider that the FA fragment must still be detected at the MS2 level.

Finally, method precision was evaluated by assessing intra- and interday variability across all tissues. The average intraday precision, expressed as RSD, was ≤5% for all ganglioside standards in all matrices (Figure 5G). Interday precision in average showed similarly robust performance, with RSD values not exceeding 10% across 3 days of analysis (Figure 5H). As depicted, it was again single standards in specific tissues that resulted in higher variations. These results demonstrate good repeatability of the method for quantitative ganglioside analysis in diverse biological matrices.

### Analysis of Gangliosides in Mouse Tissues

After evaluation of method performance across all ten mouse tissues, the developed method was applied to determine ganglioside concentrations in each tissue. While gangliosides are known to be highly abundant in brain tissue,^11,34^ their distribution and molecular species composition in other murine tissues remain considerably less explored. Comprehensive profiling revealed pronounced tissue-specific differences in both ganglioside subclass composition and molecular species diversity. In total, 80 ganglioside molecular species belonging to 18 distinct ganglioside subclasses were quantified at the molecular species level across the investigated tissues (Figure 6A). The number and identity of detected gangliosides varied substantially between tissues. Brain tissue exhibited the highest molecular diversity, with 42 ganglioside species detected, including 27 species that were exclusively observed in brain. Other tissues contained fewer ganglioside species overall (ranging from 8 in kidney to 28 species in spleen) but nevertheless displayed distinct, tissue-specific ganglioside profiles. Despite the pronounced tissue specificity, a subset of gangliosides was conserved across all tissues analyzed. Notably, four GM3 species with ceramide compositions 18:1;2/16:0;0, 18:1;2/18:0;0, 18:1;2/20:0;0, and 18:1;2/24:1;0 were detected in all ten organs. Given that GM3 serves as the biosynthetic precursor for all downstream ganglioside subclasses,^4^ the ubiquitous presence of these GM3 species is consistent with their central role in ganglioside metabolism and suggests a fundamental requirement for GM3 across diverse tissue types.

**Figure 6:**
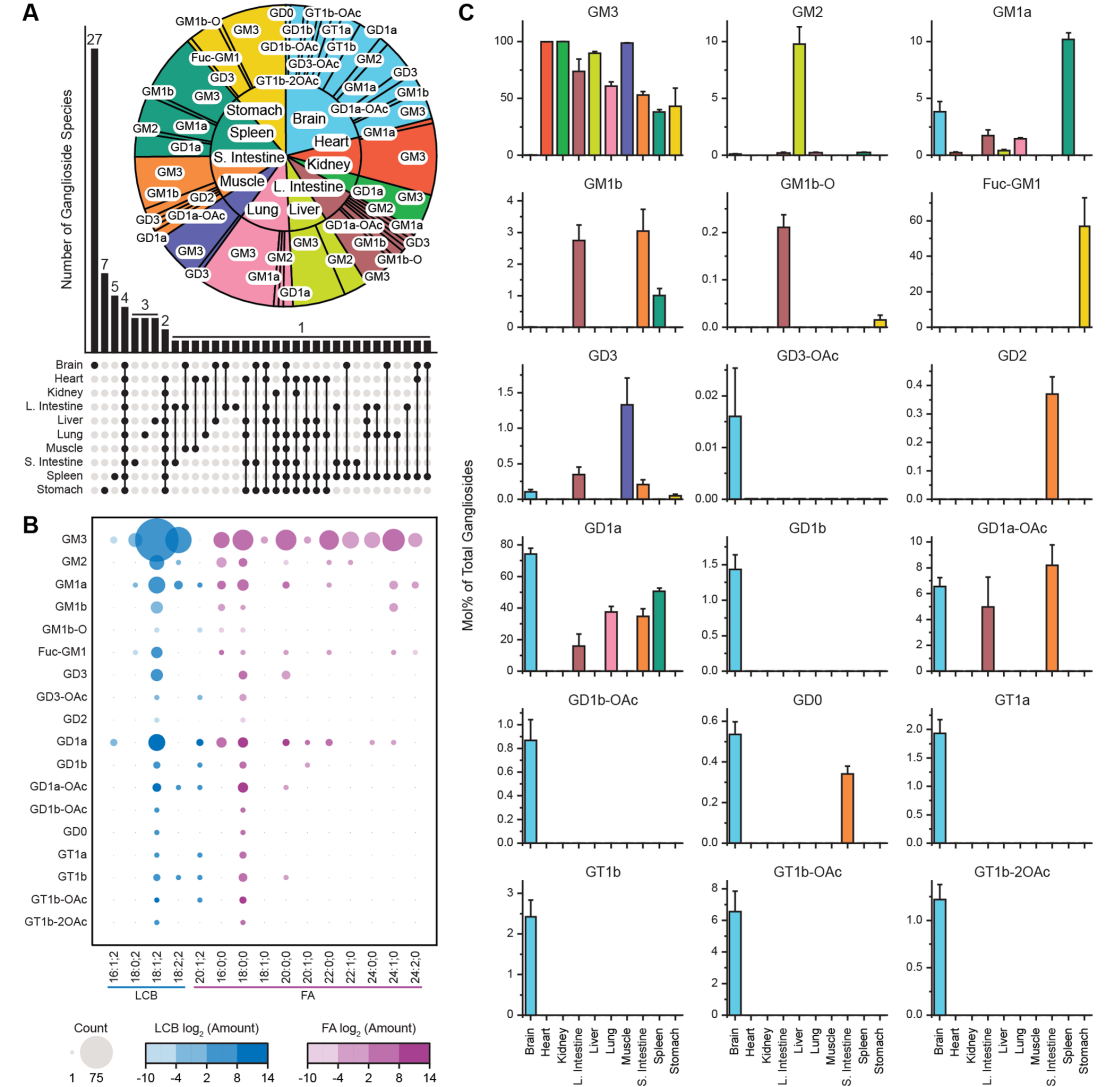
Ganglioside distribution across ten mouse tissues. (A) Number of quantified ganglioside molecular species and the distribution of shared and tissue-specific species across organs. Each column indicates the number of species identified, with black dots denoting the tissues in which the respective species were detected. The pie chart summarizes the ganglioside subclasses present in each tissue. (B) Abundance and frequency of long-chain base (LCB) and fatty acyl (FA) compositions, obtained by summing molecular species-level concentrations across all tissues. (C) Subclass-level abundances determined by summing all molecular species within each subclass and calculating mol % values per tissue.

Compared to the porcine brain extract analyzed earlier in this study, a smaller number of ganglioside subclasses was quantified across murine tissues. This difference is largely attributable to the limited sample amounts available for mouse tissues and to the stringent requirement for detecting FA-derived fragment ions for molecular species-level annotation. As a consequence, subclasses present at very low abundance and lacking detectable FA fragments were not reported. Notably, the subclasses absent from the murine data set predominantly comprised highly sialylated gangliosides containing four or five sialic acid residues, which are more readily detected in porcine brain. While methodological constraints likely contribute to this observation, the data may also reflect a lower overall complexity of the murine glycosphingolipidome compared to porcine brain tissue.

From the summed concentrations of all species across the ten organs, we determined the abundance of each LCB and FA composition (Figure 6B). As expected, species containing LCB 18:1;2 were the most abundant overall in both concentration and number.^35^ However, in brain tissue, gangliosides with LCB 18:2;2 and 20:1;2 were also present at high abundance, distributed across different subclasses. In general, LCB 18:1;2 and FA 18:0;0 were the only compositions detected in every subclass. Apart from FA 18:0;0, FA 20:0;0 was the second most abundant fatty acyl.

Next, subclass-level abundances were determined by summing all species within the same subclass and calculating mol % per tissue (Figure 6C). This analysis highlighted pronounced tissue-specific patterns. Most of the more complex subclasses were restricted to brain, with GD1a being the most abundant, followed by GT1b-OAc, GD1a-OAc, and GM1a. In all other tissues except spleen and stomach, the simplest ganglioside subclass, GM3, predominated. In spleen, GD1a was the most abundant subclass, which was also present at high levels in lung and small intestine, whereas in stomach, Fuc-GM1 was the predominant subclass. Although part of the observed tissue specificity may reflect differences in ganglioside abundance and detection sensitivity, particularly because species lacking FA-specific MS2 information were excluded, these results indicate that gangliosides, together with other glycosphingolipids, form distinct and tissue-specific molecular patterns (Figure S3). This supports the concept that ganglioside composition is tightly regulated in a tissue-dependent manner.

## Conclusion

In this study, we developed and validated a HILIC-nanoESI-MS/MS workflow that enables comprehensive, isomer-resolved analysis of gangliosides at the molecular species level. By optimizing chromatographic separation, collision energy-dependent fragmentation, and ionization conditions, the method addresses key analytical challenges in ganglioside research, including low endogenous abundance, extensive isomerism, and limited availability of analytical standards.

A central advance of the presented approach is the integration of nanoESI with online fraction collection and subsequent direct infusion, which overcomes insufficient FA fragment intensities observed when applying the conventional LC-MS/MS workflows and markedly reduces chemical background compared to standard ESI conditions. This strategy substantially increased molecular species coverage while preserving glycan isomer separation achieved by HILIC. The final method demonstrated excellent analytical performance across ten mouse tissues, including high retention time stability, low carryover, robust linearity, acceptable matrix effects, nanomolar sensitivity, and strong intra- and interday precision.

Application of the method enabled quantification of 80 gangliosides from 18 subclasses across murine tissues and revealed pronounced tissue-specific ganglioside patterns. Brain tissue exhibited the highest molecular diversity and abundance of complex subclasses, while other organs displayed distinct and characteristic profiles, highlighting the tight tissue-dependent regulation of ganglioside composition. These findings underscore the importance of considering both glycan and ceramide heterogeneity to fully understand ganglioside biology.

Overall, this work provides a powerful and versatile platform for detailed ganglioside profiling in complex biological matrices. The methodological advances described here lay the foundation for future studies exploring the functional roles of gangliosides in physiology and disease, their tissue-specific regulation, and how they change in developmental or pathological states. By enabling comprehensive, isomer-resolved quantification, this workflow opens new opportunities for uncovering the molecular complexity and biological significance of gangliosides at an unprecedented level of detail.

## Supplementary Material



## Notes

Experimental procedures, additional data, spray stability evaluation, chromatograms of porcine brain extract, and references (PDF)

## References

[1] Fahy E., Subramaniam S., Brown H. A., Glass C. K., Merrill A. H., Murphy R. C., Raetz C. R. H., Russell D. W., Seyama Y., Shaw W. (2005). A comprehensive classification
system for lipids. J. Lipid Res..

[2] Hájek R., Jirásko R., Lísa M., Cífková E., Holčapek M. (2017). Hydrophilic
Interaction Liquid Chromatography–Mass
Spectrometry Characterization of Gangliosides in Biological Samples. Anal. Chem..

[3] Moser K. L. W., Van Aken G., DeBord D., Hatcher N. G., Maxon L., Sherman M., Yao L., Ekroos K. (2021). High-defined quantitative
snapshots of the ganglioside lipidome using high resolution ion mobility
SLIM assisted shotgun lipidomics. Anal. Chim.
Acta.

[4] Kolter T. (2012). Ganglioside
Biochemistry. ISRN Biochem..

[5] Barrientos R. C., Zhang Q. (2020). Recent advances in the mass spectrometric analysis of glycosphingolipidome
– A review. Anal. Chim. Acta.

[6] Khalil M. B., Hou W., Zhou H., Elisma F., Swayne L. A., Blanchard A. P., Yao Z., Bennett S. A. L., Figeys D. (2010). Lipidomics era: Accomplishments
and challenges. Mass Spectrom. Rev..

[7] Ranasinghe A., Ciccimaro E., D’Arienzo C., Olah T. V., Ponath P., Hnatyshyn S. (2021). An integrated Qual/Quan strategy for ganglioside lipidomics
using high-resolution mass spectrometry and Skyline software. Rapid Commun. Mass Spectrom..

[8] Svennerholm L., Fredman P. (1980). A procedure
for the quantitative isolation of brain
gangliosides. Biochim. Biophys. Acta, Lipids
Lipid Metab..

[9] Folch J., Lees M., Stanley G. H. S. (1957). A simple method
for the isolation
and purification of total lipides from animal tissues. J. Biol. Chem..

[10] Bligh E. G., Dyer W. J. (1959). A rapid method of total lipid extraction
and purification. Can. J. Biochem. Physiol..

[11] Arends M., Weber M., Papan C., Damm M., Surma M. A., Spiegel C., Djannatian M., Li S., Connell L., Johannes L. (2022). Ganglioside lipidomics
of CNS myelination using
direct infusion shotgun mass spectrometry. iScience.

[12] Lee J., Yin D., Yun J., Kim M., Kim S.-W., Hwang H., Park J. E., Lee B., Lee C. J., Shin H.-S., An H. J. (2024). Deciphering mouse
brain spatial diversity via glyco-lipidomic mapping. Nat. Commun..

[13] Lydic T. A., Busik J. V., Reid G. E. (2014). A monophasic extraction
strategy
for the simultaneous lipidome analysis of polar and nonpolar retina
lipids. J. Lipid Res..

[14] Khoury S., Masson E., Sibille E., Cabaret S., Berdeaux O. (2020). Rapid sample
preparation for ganglioside analysis by liquid chromatography mass
spectrometry. J. Chromatogr. B.

[15] Popa I., Vlad C., Bodennec J., Portoukalian J. (2002). Recovery of
gangliosides from aqueous solutions on styrene-divinylbenzene copolymer
columns^1^. J. Lipid Res..

[16] Müthing, J. TLC in Structure and Recognition Studies of Glycosphingolipids. In Glycoanalysis Protocols; Hounsell, E. F. , Ed.; Humana Press, 1998; pp 183–195. 10.1385/0-89603-355-4:183 9664354

[17] Tanabe A., Matsuda M., Fukuhara A., Miyata Y., Komuro R., Shimomura I., Tojo H. (2009). Obesity causes a shift in metabolic
flow of gangliosides in adipose tissues. Biochem.
Biophys. Res. Commun..

[18] Hohenwallner K., Troppmair N., Panzenboeck L., Kasper C., El Abiead Y., Koellensperger G., Lamp L. M., Hartler J., Egger D., Rampler E. (2022). Decoding Distinct Ganglioside Patterns of Native and
Differentiated Mesenchymal Stem Cells by a Novel Glycolipidomics Profiling
Strategy. JACS Au.

[19] O’Brien J. P., Brodbelt J. S. (2013). Structural Characterization
of Gangliosides and Glycolipids
via Ultraviolet Photodissociation Mass Spectrometry. Anal. Chem..

[20] Barrientos R. C., Vu N., Zhang Q. (2017). Structural
Analysis of Unsaturated Glycosphingolipids
Using Shotgun Ozone-Induced Dissociation Mass Spectrometry. J. Am. Soc. Mass Spectrom..

[21] Ledeen R. W., Wu G., André S., Bleich D., Huet G., Kaltner H., Kopitz J., Gabius H.-J. (2012). Beyond glycoproteins as galectin
counterreceptors: tumor-effector T cell growth control via ganglioside
GM1. Ann. N.Y. Acad. Sci..

[22] Uncini A. (2012). A common mechanism
and a new categorization for anti-ganglioside antibody-mediated neuropathies. Exp. Neurol..

[23] Hakomori S. (2004). Carbohydrate-to-carbohydrate
interaction, through glycosynapse, as a basis of cell recognition
and membrane organization. Glycoconjugate J..

[24] Cantù L., Del Favero E., Sonnino S., Prinetti A. (2011). Gangliosides and the
multiscale modulation of membrane structure. Chem. Phys. Lipids.

[25] Maula T., Artetxe I., Grandell P.-M., Slotte J. P. (2012). Importance of the
Sphingoid Base Length for the Membrane Properties of Ceramides. Biophys. J..

[26] Hirano K., Kinoshita M., Matsumori N. (2022). Impact of sphingomyelin acyl chain
heterogeneity upon properties of raft-like membranes. Biochim. Biophys. Acta, Biomembr..

[27] Coman C., Solari F. A., Hentschel A., Sickmann A., Zahedi R. P., Ahrends R. (2016). Simultaneous Metabolite,
Protein, Lipid Extraction
(SIMPLEX): A Combinatorial Multimolecular Omics Approach for Systems
Biology. Mol. Cell. Proteomics.

[28] Adams K. J., Pratt B., Bose N., Dubois L. G., St John-Williams L., Perrott K. M., Ky K., Kapahi P., Sharma V., MacCoss M. J. (2020). Skyline for Small Molecules: A Unifying Software
Package for Quantitative Metabolomics. J. Proteome
Res..

[29] Herzog R., Schuhmann K., Schwudke D., Sampaio J. L., Bornstein S. R., Schroeder M., Shevchenko A. (2012). LipidXplorer: A Software for Consensual
Cross-Platform Lipidomics. PLoS One.

[30] Herzog R., Schwudke D., Schuhmann K., Sampaio J. L., Bornstein S. R., Schroeder M., Shevchenko A. (2011). A novel informatics concept for high-throughput
shotgun lipidomics based on the molecular fragmentation query language. Genome Biol..

[31] Berthold, M. R. ; Cebron, N. ; Dill, F. ; Gabriel, T. R. ; Kötter, T. ; Meinl, T. ; Ohl, P. ; Sieb, C. ; Thiel, K. ; Wiswedel, B. KNIME: The Konstanz Information Miner. In Data Analysis, Machine Learning and Applications. Studies in Classification, Data Analysis, and Knowledge Organization; Preisach, C. ; Burkhardt, H. ; Schmidt-Thieme, L. ; Decker, R. , Eds.; Springer: Berlin, Heidelberg, 2008; pp 319–326 10.1007/978-3-540-78246-9_38.

[32] Vo H. G., Gonzalez-Escamilla G., Mirzac D., Rotaru L., Herz D., Groppa S., Bindila L. (2025). Extended coverage of human serum
glycosphingolipidome by 4D-RP-LC TIMS-PASEF unravels association with
Parkinson’s disease. Nat. Commun..

[33] Wang M., Wang C., Han X. (2017). Selection of internal standards for
accurate quantification of complex lipid species in biological extracts
by electrospray ionization mass spectrometry-What, how and why?. Mass Spectrom. Rev..

[34] Borgmeyer M., Coman C., Has C., Schött H.-F., Li T., Westhoff P., Cheung Y. F. H., Hoffmann N., Yuanxiang P., Behnisch T. (2021). Multiomics
of synaptic junctions reveals altered
lipid metabolism and signaling following environmental enrichment. Cell Rep..

[35] Muralidharan S., Shimobayashi M., Ji S., Burla B., Hall M. N., Wenk M. R., Torta F. (2021). A reference map of
sphingolipids
in murine tissues. Cell Rep..

