# Supplementary material for: Comprehensive Isomer-Resolved Ganglioside Profiling by HILIC-nanoESI-MS/MS
Source: Anal Chem. 2026 Jul 6;98(30):22068–79. doi: 10.1021/acs.analchem.6c00380 (PMC13449219; doi:10.1021/acs.analchem.6c00380)
Supplement: Supplementary file 1 [file ac6c00380_si_001.pdf]

Supporting Information

Comprehensive isomer-resolved ganglioside profiling by HILIC-nanoESI-MS/MS

Nina Troppmair[1,2], Jonas Santol[3,4,5], Mark Baumert[6], Lukáš Opálka[7], Alice Assinger[3,8], Cristina Coman[1*], Robert Ahrends[1*]

[1] Department of Analytical Chemistry, Faculty of Chemistry, University of Vienna, 1090 Vienna, Austria

[2] Vienna Doctoral School in Chemistry, University of Vienna, 1090 Vienna, Austria

[3] Department of Vascular Biology and Thrombosis Research, Center of Physiology and Pharmacology, Medical University of Vienna, 1090 Vienna, Austria

[4] Department of Surgery, HPB Center, Vienna Health Network, Clinic Favoriten and Sigmund Freud Private University, 1100 Vienna, Austria

[5] Division of Hepatobiliary and Pancreas Surgery, Department of Surgery, Mayo Clinic, Rochester, MN 55905, USA

[6] Advion Ltd, Harlow Enterprise Hub, Harlow CM20 2NQ, United Kingdom

[7] Department of Organic and Bioorganic Chemistry, Faculty of Pharmacy, Charles University, 500 03 Hradec Králové, Czech Republic

[8] Ludwig Boltzmann Institute of Cardiovascular Research, 1090 Vienna, Austria

* Email: cristina.coman@univie.ac.at and robert.ahrends@univie.ac.at

Table of contents

EXPERIMENTAL PROCEDURES 2
Text S1. Materials. 2
Text S2. Samples. 2
Text S3. Sample Preparation. 2
Text S4. LC-nanoESI-MS/MS. 3
Text S5. Direct Infusion MS and MS/MS of Fractions. 3
Text S6. LC-ESI-MS/MS. 4
Text S7. LC-MS/MS Analysis of Other Sphingolipids. 4
Text S8. Column Comparison. 4
Text S9. Internal standards and quantification. 5
Text S10. Method validation. 5
FIGURES 6
Figure S1. Adduct formation of ganglioside standards under different solvent conditions. 6
Figure S2. Evaluation of spray stability across different nanoESI chips. 7
Figure S3. Dynamic range of the sphingolipid lipidome across ten mouse tissues. 9
REFERENCES 10

EXPERIMENTAL PROCEDURES

Text S1. Materials. Chemicals and lipid standards were obtained from the following sources: Ammonium acetate, ammonium formate, acetic acid, phosphoric acid, tert-butyl methyl ether (MTBE), and chloroform were obtained from Sigma-Aldrich (Steinheim, Germany). Acetonitrile (ACN), methanol (MeOH), water, and formic acid were purchased from Biosolve (Valkenswaard, The Netherlands), isopropanol (IPA) from Honeywell (Seelze, Germany), and 1-butanol (1-BuOH) from Merck (Darmstadt, Germany). All solvents were MS-grade. Ceramide/Sphingoid Internal Standard Mixture II (containing sphingosine 17:1;2, sphinganine 17:0;2, sphingosine-1-P 17:1;2, sphinganine-1-P 17:0;2, sphingomyelin 18:1;2/12:0;0, ceramide 18:1;2/12:0;0, glucosylceramide 18:1;2/12:0;0, lactosylceramide 18:1;2/12:0;0, and ceramide-1-P 18:1;2/12:0;0), mono-sulfo galactosyl(ß) ceramide 18:1;2/12:0;0, D7-GM3 18:1;2/18:0;0, D7-GM2 18:1;2/18:0;0, D7-GM1a 18:1;2/18:0;0, D7-GD3 18:1;2/18:0;0, D7-GD2 18:1;2/18:0;0, D7-GD1b 18:1;2/18:0;0, D7-Fuc-GM1 18:1;2/18:0;0, GT1b 18:1;2/18:0;0 as well as the total ganglioside extract from porcine brain were from Avanti Polar Lipids (Alabaster, AL, USA).

Text S2. Samples. Four male C57BL/6J mice were euthanized in accordance with institutional and national guidelines for animal welfare by intraperitoneal injection of ketamine (250 mg/kg) and xylazine (25 mg/kg). Following confirmation of death, tissues were immediately excised, rinsed several times using phosphate-buffered saline (PBS), snap-frozen in liquid nitrogen, and stored at -80 °C until further processing. From each mouse, ten tissues were collected namely brain, heart, kidney, large intestine, liver, lung, muscle, small intestine, spleen, and stomach.
For comparison of the conventional ESI and nanoESI setups, approximately 15 µg of total porcine brain extract were dissolved in 1 mL of solvent and used for the respective measurements.

Text S3. Sample Preparation. Lipid extraction was carried out based on the method described by Coman *et al.*[1] with slight modifications. Briefly, 225 µL of MeOH and the internal standard mixture were added to each sample, followed by ultrasonication in a water bath with ice. Subsequently, 750 µL of MTBE were added and the samples were incubated for 1 h at 4 °C with agitation at 950 rpm. Phase separation was initiated by the addition of 188 µL of water, after which samples were centrifuged for 10 min at 10 000 g and 4 °C. The upper organic phase was carefully collected and evaporated to dryness under a stream of nitrogen. Dried samples were reconstituted in 1-BuOH/IPA/H_2O (8/23/69, v/v/v) containing 5 mM phosphoric acid.[2] To precipitate proteins from the remaining aqueous phase, 527 µL of MeOH were added, and samples were incubated at -20 °C for 2 h, followed by centrifugation for 30 min at 13 500 g. The resulting supernatant containing the gangliosides was collected, dried, and reconstituted in ACN/MeOH (3/1, v/v) prior to analysis.

Text S4. LC-nanoESI-MS/MS. The following gradient was applied: initial 80.0% B, 55.0% B at 6.5 min, held at 55.0% B until 8.5 min, 40.0% B at 11.5 min, held at 40.0% B until 13.5 min, 80.0% B from 13.6 to 22.0 min.

Each fraction was collected for 6 s, resulting in a total volume of 20 µL per well. In the example shown in **Figure 4F**, fractions were collected between 2.3 and 7.1 min, while the flow was directed to waste during the remainder of the LC run. To ensure compatibility with direct infusion measurements, the solvent composition at the time of fraction collection was compared to the solvent commonly used for shotgun experiments (IPA/MeOH/$CHCl_3$, 4/2/1, v/v/v, containing 7.5 mM ammonium acetate) and gave comparable results.

The ion transfer tube was maintained at 350 °C, with the S-lens set to 70%. The 'Mild Trapping' option was enabled to minimize unintended precursor ion dissociation. The MS method was segmented into two time-dependent acquisition windows featuring tailored full-scan m/z ranges and isolation widths matched to the elution of specific ganglioside subclasses. The first method used a scan range of 800 – 1400 m/z, while for the second one it was 600 – 1200 m/z. For all of them a resolution of 60 000, an AGC target of 10^6 and a maximum IT of 105 ms. The MS1 scan was followed by a data dependent acquisition (DDA) of the 20 most intense signals. The isolation window was 1 m/z for the first method and 0.5 m/z for the second one. MS/MS spectra were acquired with a resolution of 15 000, an AGC target of 10^5 and an automatic maximum IT.

Text S5. Direct Infusion MS and MS/MS of Fractions. Direct infusion measurements were performed with an ionization voltage of -1.3 kV and a back pressure of 0.9 psi. The ion transfer capillary temperature was set to 250 °C, and the S-lens level was adjusted to 70%. To minimize unintended precursor ion fragmentation, the 'Mild Trapping' mode was enabled. Full-scan MS acquisition employed fraction-specific, narrowed m/z ranges, with isolation widths adapted accordingly. MS1 spectra were acquired at a resolution of 240 000 using an AGC target of 10^6 and a maximum injection time of 105 ms. MS/MS spectra were recorded at a resolution of 60 000 with an AGC target of 10^5 and a maximum injection time of 105 ms.

The raw data were converted using msConvert (version 3.0.25190-0392f93).[3] Mass spectra were processed with LipidXplorer (version 1.2.9) [4, 5] and compiled into a MasterScan database using a mass tolerance of 5 ppm. The m/z ranges were adjusted depending on the analyzed fraction, as described above, with intensity thresholds of 7.5×10^3 for MS and 4.5×10^3 for MS/MS data. Lipid species were assigned by matching monoisotopic precursor masses to their corresponding elemental compositions. Identification confidence was further increased by applying self-written molecular fragmentation query language (MFQL) queries designed to detect precursor ions together with their characteristic fragment ions. The resulting output files were subsequently processed using a custom-developed R script (RStudio version 2024.12.0+467; RStudio Team).

Text S6. LC-ESI-MS/MS. For LC-ESI-MS/MS measurements except for the following parameters everything was kept identical as described for the LC-nanoESI-MS/MS acqusitions. The ion transfer capillary temperature was maintained at 300 °C, the vaporizer temperature at 340 °C, and the spray voltage at -2.5 kV. The acquisition method was divided into three time-resolved segments, each optimized with subclass-specific full-scan m/z ranges and isolation widths corresponding to ganglioside elution. The first and second segments covered an m/z range of 700 – 1500, whereas the third segment spanned 600 – 1400 m/z. Isolation widths were set to 1 m/z for the first segment and narrowed to 0.5 m/z for the second and third segments.

Text S7. LC-MS/MS Analysis of Other Sphingolipids. Analysis of additional sphingolipid classes was performed with minor modifications to a previously reported method by Peng *et al*.[6] and Troppmair *et al.*[7] using a Vanquish Flex UHPLC system (Thermo Fisher Scientific, Germering, Germany) coupled to a QTRAP 6500+ mass spectrometer (AB Sciex, Darmstadt, Germany). For each sample, 5 µL were injected onto an Ascentis Express C18 column (150 × 2.1 mm, 2.7 µm; Supelco, Bellefonte, PA, USA) equipped with a corresponding guard cartridge (50 × 2.1 mm, 2.7 µm; Supelco). The autosampler was maintained at 10 °C, while the column oven was set to 60 °C. Mobile phase A consisted of ACN/H_2O (60/40, v/v), and mobile phase B of IPA/ACN (90/10, v/v), both supplemented with 10 mM ammonium formate, 0.1% formic acid, and 5 µM phosphoric acid. Chromatographic separation was achieved using a 25 min gradient at a flow rate of 0.5 mL/min. Prior to each injection, the injector needle was automatically washed with 30% mobile phase B. The QTRAP instrument was operated in positive ion mode using a Turbo V electrospray ionization source. Source parameters were as follows: curtain gas 30 psi, collision gas medium, ion spray voltage +5500 V, source temperature 250 °C, ion source gas 1 at 40 psi, ion source gas 2 at 65 psi, declustering potential +75 V, entrance potential +10 V, and exit potential +17 V. Scheduled multiple reaction monitoring (MRM) was employed with both Q1 and Q3 operated at unit resolution, a retention time window of 2 min, and a scan time of 0.5 s.
Transition lists were generated using LipidCreator (version 1.1.0.736).[8] Data acquisition for targeted sphingolipid analysis was performed using Analyst software (version 1.7.2; AB Sciex). Data visualization and manual peak integration were carried out in Skyline (version 25.1.0.142).[9] Post-acquisition data processing was automated using Konstanz Information Miner (KNIME; version 5.2.5).[10]

Text S8. Column Comparison. In addition to the HILIC column used in the final method, three alternative columns were evaluated during method development. First, an ACQUITY UPLC BEH Amide column (150 × 2.1 mm, 1.7 µm; Waters, Wexford, Ireland) equipped with an ACQUITY UPLC BEH Amide VanGuard pre-column (5 × 2.1 mm, 1.7 µm; Waters) was tested. Although this stationary phase is based on the same amide chemistry as the final column, it lacks an inert surface treatment. As shown in **Figure 2C-D**, use of this column resulted in degradation of the ganglioside precursor ions. Two

additional columns featuring inert surface chemistries were also assessed, namely a SeQuant ZIC-pHILIC column (150 × 2.1 mm, 5 µm; Supelco, Bellefonte, PA, USA) with a matching guard column (20 × 2.1 mm, 5 µm; Supelco), and an iHILIC-(P) Classic column (100 × 2.1 mm, 5 µm; HILICON, Umeå, Sweden) equipped with an iHILIC-(P) Classic guard column (20 × 2.1 mm, 5 µm; HILICON). Both columns successfully prevented precursor modification, however, neither provided sufficient chromatographic resolution to separate ganglioside isomers adequately.

Text S9. Internal standards and quantification. Seven deuterated ganglioside standards, together with the internal standards (IS) used for the other sphingolipid classes described above, were employed to normalize endogenous analyte abundances. For ganglioside subclasses lacking a dedicated internal standard, the most structurally similar available standard was used for quantification. The amounts of internal standards added were chosen to fall within an appropriate range relative to the expected analyte concentrations in the biological matrices, considering the overall dynamic range.

Text S10. Method validation. The developed HILIC-nanoESI-MS/MS method was systematically validated to assess its suitability for quantitative ganglioside analysis in complex biological matrices based on the ICH guideline.[11]

Retention Time Stability. The stability of the retention time was assessed across seven ganglioside standards in ten mouse tissues. Samples were analyzed on four different days, and retention time stability was expressed as relative standard deviation (RSD).

Carryover. Carryover was evaluated by injecting blank samples immediately after the highest calibrator for each ganglioside standard and tissue matrix. Carryover was calculated as the ratio (%) of the signal observed in the blank to that of the preceding calibrator injection.

Linearity. Calibration curves were generated for each of the seven ganglioside standards by spiking six concentration levels (10, 50, 100, 250, 500, 1000 nM) into the tissue matrices. Linearity was assessed using weighted linear regression, calculating the coefficients of determination (R^2).

Matrix Effects. Matrix effects were evaluated by comparing the slopes of calibration curves prepared in biological matrices with those obtained in neat solvent. Matrix effects were expressed as the ratio of the slopes (matrix/solvent).

Sensitivity. Method sensitivity was characterized by determining limits of detection (LOD) and lower limits of quantification (LLOQ) for all ganglioside standards in each tissue. LOD and LLOQ were defined based on signal-to-noise ratios of 3:1 and 5:1, respectively.

Precision. Intraday precision was evaluated by repeated analysis of three spiked samples within a single analytical run, while interday precision was assessed across 3 independent days. Precision was expressed as RSD of quantified concentrations.

FIGURES

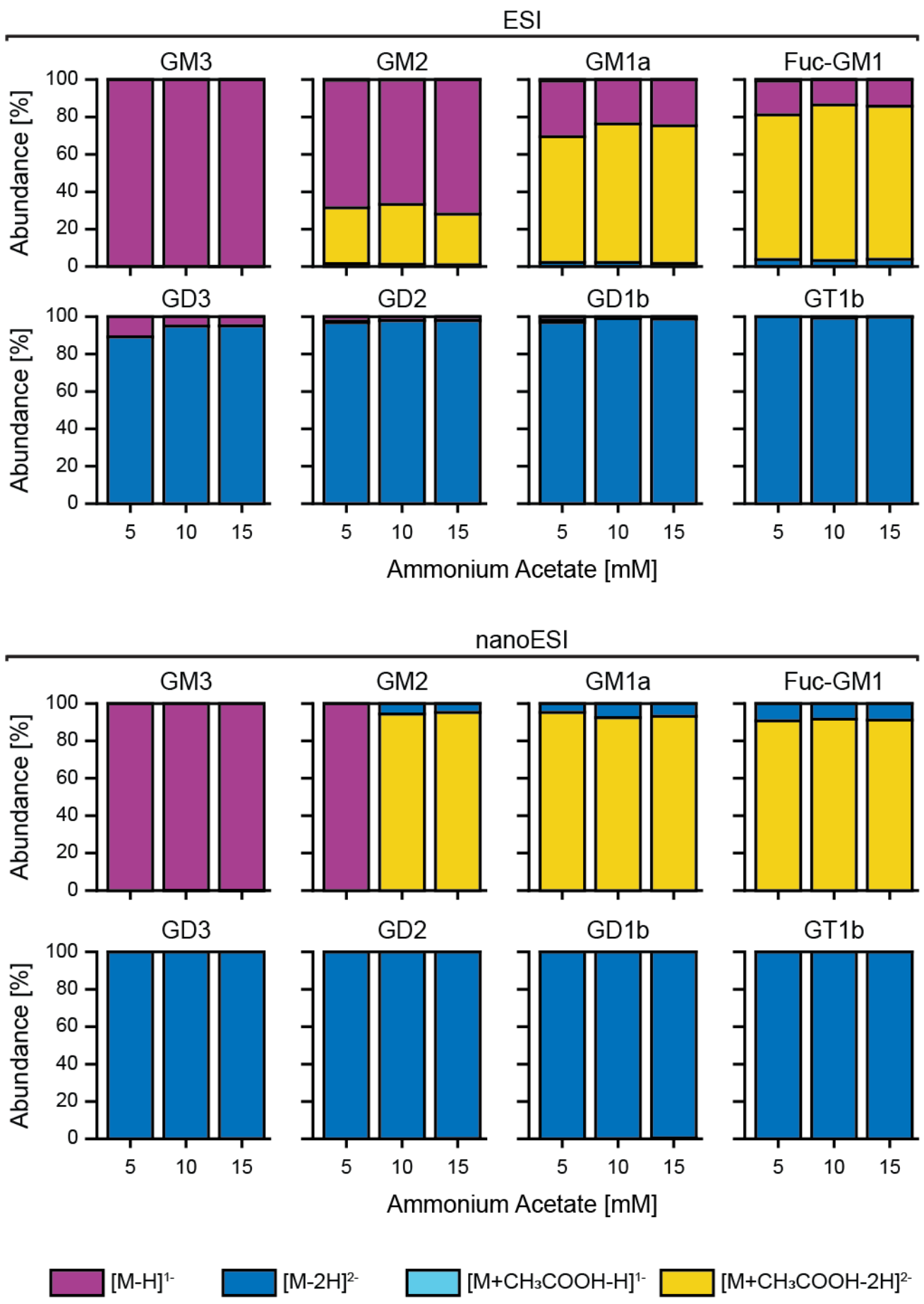

Figure S1. Adduct formation of ganglioside standards under different solvent conditions. Relative distribution of adduct species for eight ganglioside standards analyzed using conventional ESI and nanoESI sources at three ammonium acetate concentrations (5 mM, 10 mM, and 15 mM). Data are shown as normalized peak areas.

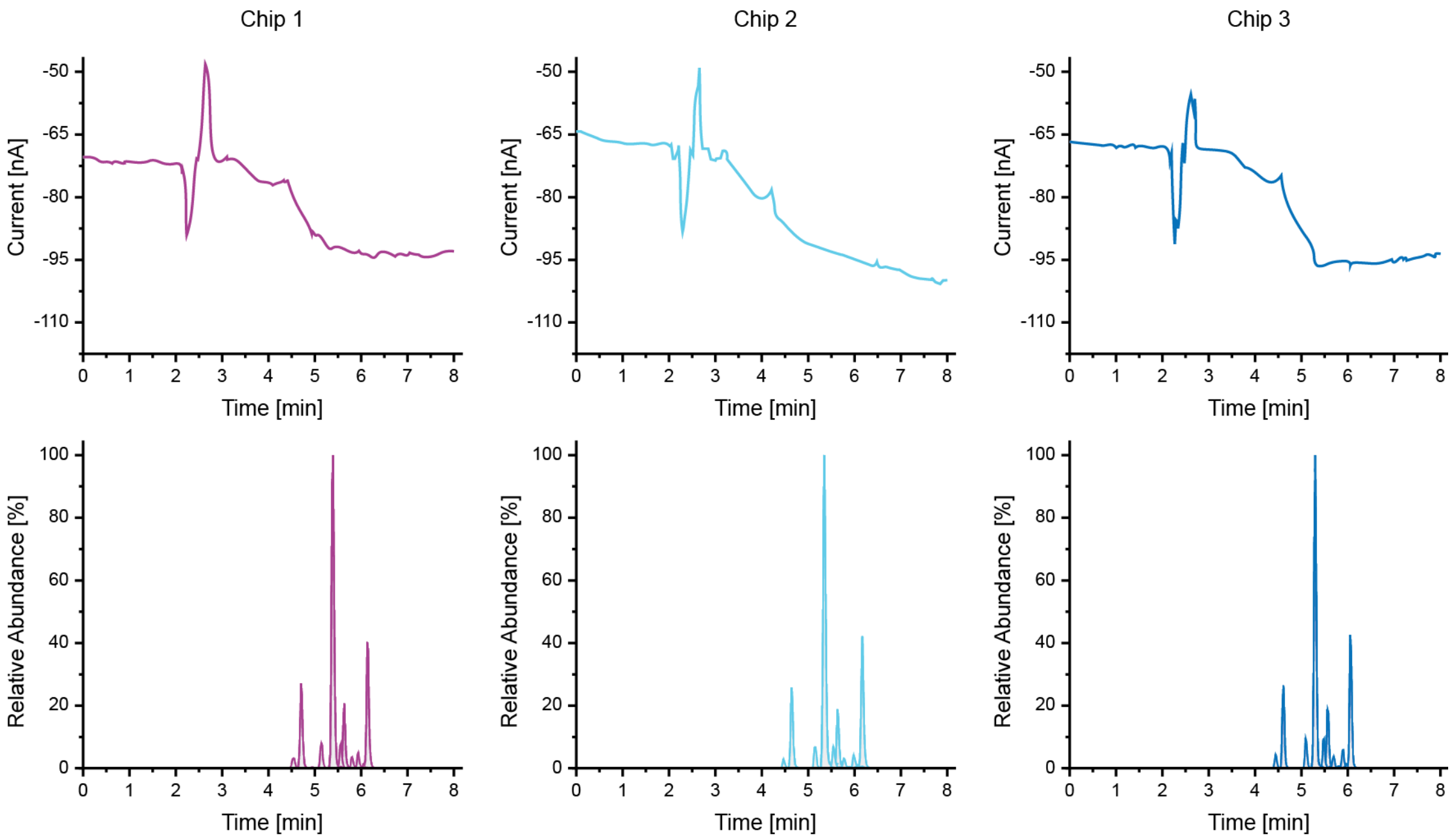

Figure S2. Evaluation of spray stability across different nanoESI chips. Spray current traces and corresponding chromatograms from replicate injections of the porcine brain extract acquired using three chips from different batches. Comparable spray currents and chromatographic profiles demonstrate consistent spray performance across chips.

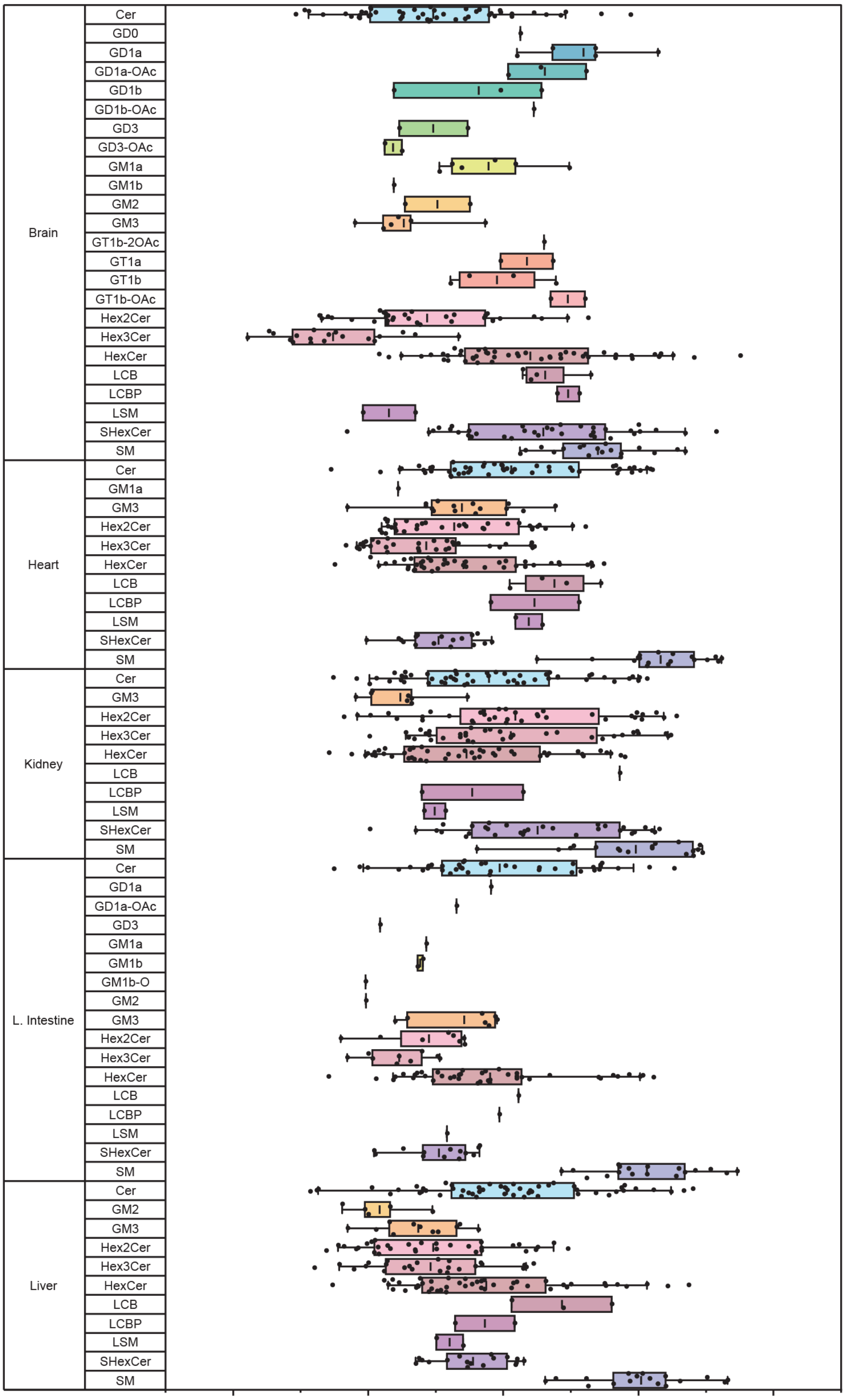

Figure S3. Continued on next page.

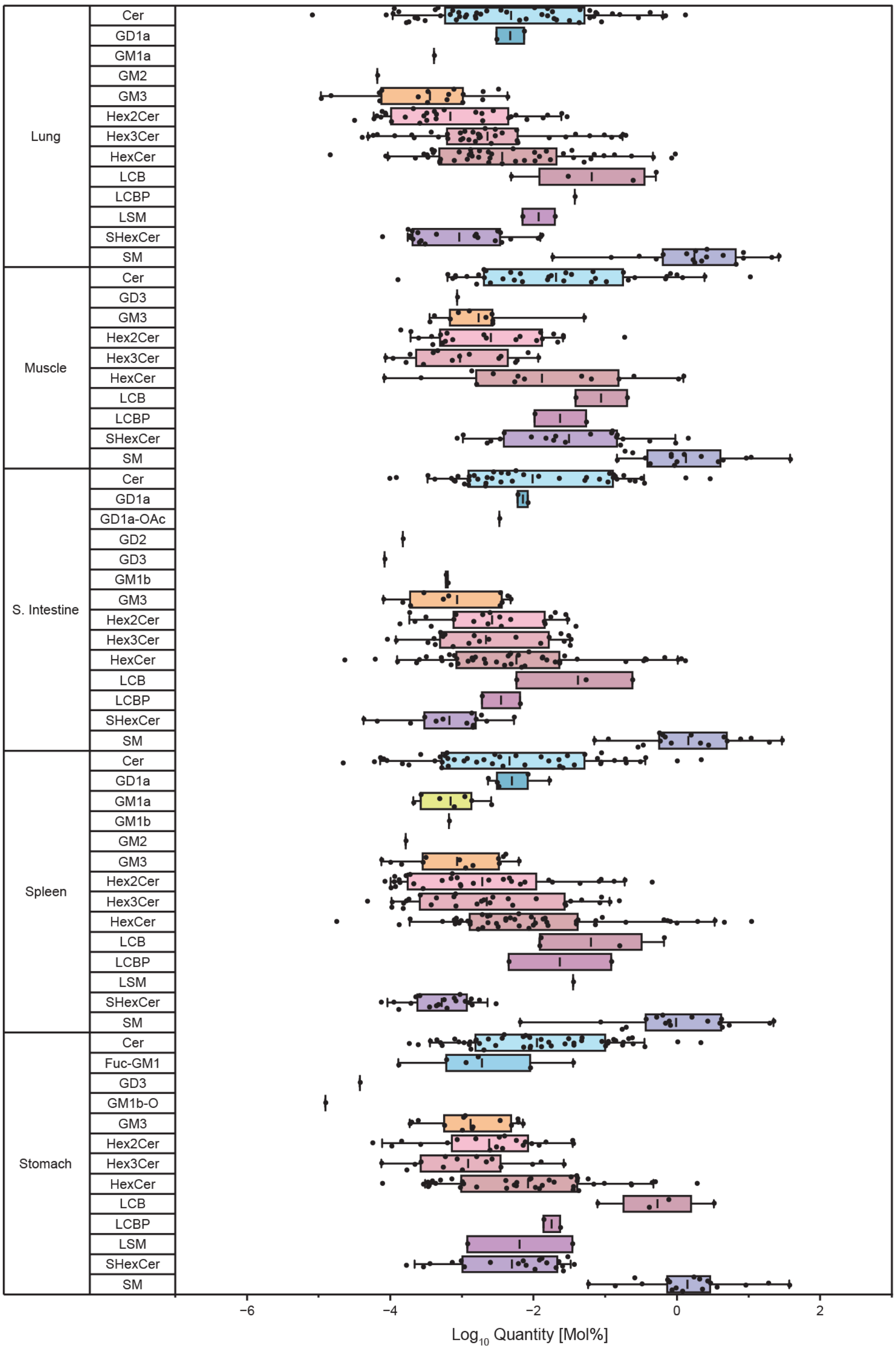

Figure S3. Dynamic range of the sphingolipid lipidome across ten mouse tissues. Boxplots showing the distribution of sphingolipid abundances in each tissue, expressed as $\log_{10}$-transformed molar percentages (mol%). Each dot represents an individual sphingolipid species. Boxes indicate the mean and 25th - 75th percentiles, while whiskers represent the 5th - 95th percentiles.

REFERENCES

(1) Coman, C.; Solari, F. A.; Hentschel, A.; Sickmann, A.; Zahedi, R. P.; Ahrends, R. Simultaneous Metabolite, Protein, Lipid Extraction (SIMPLEX): A Combinatorial Multimolecular Omics Approach for Systems Biology. *Molecular & cellular proteomics* 2016, *15* (4), 1435-1466. DOI: 10.1074/mcp.M115.053702.

(2) Danne-Rasche, N.; Coman, C.; Ahrends, R. Nano-LC/NSI MS Refines Lipidomics by Enhancing Lipid Coverage, Measurement Sensitivity, and Linear Dynamic Range. *Analytical Chemistry* 2018, *90* (13), 8093–8101. DOI: 10.1021/acs.analchem.8b01275.

(3) Holman, J. D.; Tabb, D. L.; Mallick, P. Employing ProteoWizard to Convert Raw Mass Spectrometry Data. *Current Protocols in Bioinformatics* 2014, *46* (1), 13.24.11-13.24.19. DOI: 10.1002/0471250953.bi1324s46.

(4) Herzog, R.; Schuhmann, K.; Schwudke, D.; Sampaio, J. L.; Bornstein, S. R.; Schroeder, M.; Shevchenko, A. LipidXplorer: A Software for Consensual Cross-Platform Lipidomics. *PLoS One* 2012, *7*, e29851. DOI: 10.1371/journal.pone.0029851.

(5) Herzog, R.; Schwudke, D.; Schuhmann, K.; Sampaio, J. L.; Bornstein, S. R.; Schroeder, M.; Shevchenko, A. A novel informatics concept for high-throughput shotgun lipidomics based on the molecular fragmentation query language. *Genome Biology* 2011, *12* (1). DOI: 10.1186/gb-2011-12-1-r8.

(6) Peng, B.; Weintraub, S. T.; Coman, C.; Ponnaiyan, S.; Sharma, R.; Tews, B.; Winter, D.; Ahrends, R. A Comprehensive High-Resolution Targeted Workflow for the Deep Profiling of Sphingolipids. *Analytical Chemistry* 2017, *89* (22), 12480-12487. DOI: 10.1021/acs.analchem.7b03576.

(7) Troppmair, N.; Kopczynski, D.; Assinger, A.; Lehmann, R.; Coman, C.; Ahrends, R. Accurate Sphingolipid Quantification Reducing Fragmentation Bias by Nonlinear Models. *Analytical Chemistry* 2023, *95* (41), 15227-15235. DOI: 10.1021/acs.analchem.3c02445.

(8) Peng, B.; Kopczynski, D.; Pratt, B. S.; Ejsing, C. S.; Burla, B.; Hermansson, M.; Benke, P. I.; Tan, S. H.; Chan, M. Y.; Torta, F.; et al. LipidCreator workbench to probe the lipidomic landscape. *Nature Communications* 2020, *11*, 2057. DOI: 10.1038/s41467-020-15960-z.

(9) Adams, K. J.; Pratt, B.; Bose, N.; Dubois, L. G.; St John-Williams, L.; Perrott, K. M.; Ky, K.; Kapahi, P.; Sharma, V.; MacCoss, M. J.; et al. Skyline for Small Molecules: A Unifying Software Package for Quantitative Metabolomics. *J Proteome Res* 2020, *19* (4), 1447-1458. DOI: 10.1021/acs.jproteome.9b00640 PubMed.

(10) Berthold, M. R.; Cebron, N.; Dill, F.; Gabriel, T. R.; Kötter, T.; Meinl, T.; Ohl, P.; Sieb, C.; Thiel, K.; Wiswedel, B. KNIME: The Konstanz Information Miner. In *Data Analysis, Machine Learning and Applications. Studies in Classification, Data Analysis, and Knowledge Organization.*, 2008; Preisach, C., Burkhardt, H., Schmidt-Thieme, L., Decker, R., Eds.; Springer, Berlin, Heidelberg: pp 319-326. DOI: 10.1007/978-3-540-78246-9_38.

(11) Committee for Medicinal Products for Human Use. ICH guideline M10 on bioanalytical method validation and study sample analysis. European Medicines Agency, Ed.; 2022.
